# Do knee abduction kinematics and kinetics predict future anterior cruciate ligament injury risk? A systematic review and meta-analysis of prospective studies

**DOI:** 10.1186/s12891-020-03552-3

**Published:** 2020-08-20

**Authors:** Anna Cronström, Mark W. Creaby, Eva Ageberg

**Affiliations:** 1 Department of Health Sciences, Lund University, Box 157, 221 00 Lund, Sweden; 2 Department of Community Medicine and Rehabilitation, Umeå University, Umeå, Sweden; 3 School of Exercise Science, Australian Catholic University, Brisbane, Australia

**Keywords:** Anterior cruciate ligament, Knee abduction, Risk factor, Knee injury

## Abstract

**Background:**

To systematically review the association between knee abduction kinematics and kinetics during weight-bearing activities at baseline and the risk of future anterior cruciate ligament (ACL) injury.

**Methods:**

Systematic review and meta-analysis according to PRISMA guidelines. A search in the databases MEDLINE (PubMed), CINAHL, EMBASE and Scopus was performed. Inclusion criteria were prospective studies including people of any age, assessing baseline knee abduction kinematics and/or kinetics during any weight-bearing activity for the lower extremity in individuals sustaining a future ACL injury and in those who did not.

**Results:**

Nine articles were included in this review. Neither 3D knee abduction angle at initial contact (Mean diff: -1.68, 95%CI: − 4.49 to 1.14, ACL injury *n* = 66, controls *n* = 1369), peak 3D knee abduction angle (Mean diff: -2.17, 95%CI: − 7.22 to 2.89, ACL injury *n* = 25, controls *n* = 563), 2D peak knee abduction angle (Mean diff: -3.25, 95%CI: − 9.86 to 3.36, ACL injury *n* = 8, controls *n* = 302), 2D medial knee displacement (cm; Mean diff:: -0.19, 95%CI: − 0,96 to 0.38, ACL injury *n* = 72, controls *n* = 967) or peak knee abduction moment (Mean diff:-10.61, 95%CI: - 26.73 to 5.50, ACL injury *n* = 54, controls *n* = 1330) predicted future ACL injury.

**Conclusion:**

Contrary to clinical opinion, our findings indicate that knee abduction kinematics and kinetics during weight-bearing activities may not be risk factors for future ACL injury. Knee abduction of greater magnitude than that observed in the included studies as well as factors other than knee abduction angle or moment, as possible screening measures for knee injury risk should be evaluated in future studies.

## Background

Anterior cruciate ligament (ACL) injury is a common injury in team sports [1, 2] and often leads to serious consequences for the individual, including pain, functional limitations, reduced quality of life and lower activity levels [3, 4] that may persist several years post injury [4]. There is also an increased risk of developing early-onset osteoarthritis of the knee [5].

Most ACL injuries occur during non-contact episodes [6], typically within 50 milliseconds after foot contact, with the foot planted on the ground with a nearly extended knee together with trunk lean and knee abduction [7, 8]. The main function of the ACL is to provide mechanical stability to the knee during movements by preventing anterior tibial translation and rotational load [9, 10]. Several in vitro studies have also shown that the knee abduction moment is a major contributor to ACL strain and is, thus, suggested to play an important role in the ACL injury mechanism [11–13]. Added to this, some studies have reported individuals with ACL deficiency to exhibit an increased knee abduction angle compared to non-injured individuals [14–16]. This, together with the result of one early study establishing a relationship between increased knee abduction angle and knee abduction moment, respectively, and a higher risk of ACL injury in women [17], have given rise to knee abduction being widely accepted as an undesirable movement pattern [6, 8]. That women are reported to perform functional tasks with greater knee abduction than men [18], as well as having a higher risk of sustaining an ACL injury [1] has further perpetuated this hypothesis.

Based on the evidence-based reasoning presented above, numerous studies have been conducted to (1) determine the factors that contribute to knee abduction during weight-bearing activities [19], and (2) to incorporate exercises to reduce knee abduction into ACL injury prevention programs [6, 8]. However, the evidence for an association between knee abduction kinematics and/or kinetics and ACL injury risk seems to be conflicting [17, 20] and to date the findings from all studies investigating knee abduction as a risk factor for future ACL injury have not been synthesized. Thus, the aim of this study was to systematically review knee abduction kinematics and kinetics during weight-bearing activities at baseline as a possible risk factor for future ACL injury development.

## Methods

A systematic review and meta-analyses were conducted according to the PRISMA guidelines. The study protocol was pre-registered (PROSPERO CRD42017067254; n.b., knee abduction kinetics were added to the protocol after registration).

### Literature search and study selection

#### Search strategy

A systematic search in MEDLINE (PubMed), CINAHL, EMBASE and Scopus was performed in September 2018 and updated in August 2019 using the terms as follows:

(“Anterior Cruciate Ligament”[Mesh]) OR “Anterior Cruciate Ligament Injuries”[Mesh])) OR lower extremity[Title/Abstract]) OR ACL injur*) OR Anterior cruciate ligament injur*)) AND ((risk factor*[Title/Abstract]) OR injury risk[Title/Abstract])) AND (((((((knee abduction[Title/Abstract]) OR biomechanic*[Title/Abstract]) OR mechanic*[Title/Abstract]) OR kinematic*[Title/Abstract] OR kinetic*[Title/Abstract]) OR valgus[Title/Abstract]) OR alignment[Title/Abstract]) OR displacement[Title/Abstract]).

In addition, reference lists of all relevant articles were searched for additional studies. No language or publication date restrictions were applied.

#### Eligibility criteria

Inclusion criteria were: 1) Prospective longitudinal studies, 2) including healthy men and/or women of any age, 3) assessing baseline knee abduction in degrees and/or medial knee displacement (MKD) in cm with 2D and/or 3D motion analysis, and/or by visual observation, and/or knee abduction moment during any weight-bearing activity for the lower extremity, and 4) recording of ACL injuries sustained during the follow-up period. Animal studies, in vitro studies, case studies, retrospective studies, conference abstracts, review papers, editorials and letters were excluded.

#### Data extraction and synthesis

Two researchers (AC & EA) independently screened the titles, abstracts and full papers against the inclusion/exclusion criteria. Any disagreements were resolved by a consensus discussion between AC and EA, and if required with a third researcher (MWC). The following data were extracted from the studies: Authors, publication date, number of participants, sex, age, activity level, sport, measurement tool (2D or 3D motion analysis, or visual observation), knee abduction angle in degrees or MKD in cm, knee abduction moment, time point during the movement at which an assessment was made (e.g., on contact with the ground or the peak angle or moment during the movement), functional task, follow-up period, ACL injury data and effect measure. A meta-analysis was performed if there were two or more studies that included the same outcome, e.g. assessed knee abduction kinematics with the same measurement tool (e.g., 3D motion analysis) at the same time point during the weight-bearing activity (e.g., peak knee abduction). If we could not retrieve sufficient data from a paper to determine inclusion/exclusion or for the purposes of data extraction, the authors were contacted and additional data were requested.

Comprehensive Meta-Analysis software, version 2.2.064 (Englewood, USA) was used for meta-analyses. The effect measure was calculated as the mean (SD) difference in baseline knee abduction angle in degrees at initial contact (IC) and/or peak knee abduction, in MKD in cm or in peak abduction moment (N.m.) between those who sustained an ACL injury and those who did not. A random effect model was used due to expected heterogeneity between studies, such as task, follow-up duration, gender, age, sport, and activity level. All meta-analysis and corresponding forest plots were weighted under the random effect model, taking both within study variance and between study variance (Tau^2^) into account [21]. Between-studies effect measure heterogeneity was calculated with the Q-test and expressed as I^2^-statistics. A *p*-value less than or equal to 0.05 was considered statistically significant. In addition, to evaluate the robustness of our meta-analyses, sensitivity analyses for different subgroups (i.e., sex, age, task and follow-up period) were performed when possible.

#### Quality assessment and publication bias

The checklist previously used by Cronström et.al [18, 19]. adapted from the original checklist by Downs and Black [22] was used for assessment of general methodological quality of the included studies (Online resource A, Table 1). Studies meeting the inclusion criteria were assessed for quality by two independent reviewers (AC) and (EA). Publication bias was explored using Funnel plots with trim and fill if ten or more studies were included in the meta-analysis [23, 24].

## Results

### Study selection

**Fig. 1:**
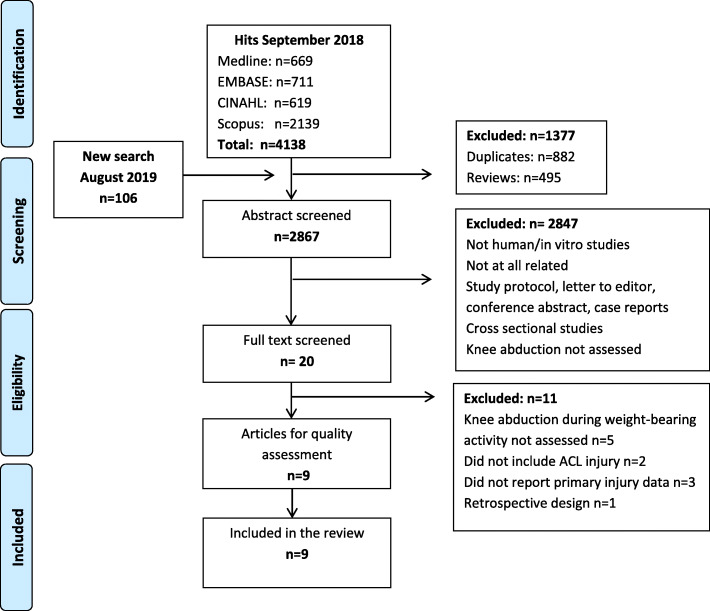
Flow chart of the inclusion process

A total of 2867 abstracts were screened against the inclusion/exclusion criteria. Twenty full-text articles were then screened. Of those, 10 articles were excluded due to not meeting the inclusion/exclusion criteria [25–34]. Of the remaining ten articles, three articles [35–37] used the total number of lower extremity injuries as an outcome and one study [38] used a combined measure of knee abduction and trunk lean. The authors of these articles were contacted and additional data for ACL injury and knee abduction specifically were requested, and were retrieved for three of the four studies [35, 37, 38]. Finally, nine articles proceeded to quality assessment and were included in the review [17, 20, 35, 37–42] (Fig. 1).

### Study characteristics

In six studies, 3D knee abduction angle was assessed at different time points; at IC (*n* = 4) [17, 20, 39, 41], peak knee abduction (*n* = 3) [17, 35, 41] or across the entire landing phase [42]. In two studies, 2D peak knee abduction angle was assessed [37, 38]. 2D MKD in cm at IC and peak was assessed in one study [40]. The 2D MKD excursion in cm (the difference between IC and peak MKD) was assessed in three studies [20, 39, 40]. Eight studies assessed knee abduction kinematics at baseline during either a double-leg vertical drop jump [17, 20, 35, 39, 41, 42] or single-leg vertical drop jump [38, 40] and one study evaluated knee abduction angle during a single-leg squat [37]. Four studies assessed peak knee abduction moment during a double-leg vertical drop jump in females [17, 20, 39, 42]. Seven studies included only females involved in either high school team sports [17, 39, 40] or playing at an elite level [20, 35, 38, 42] and two studies included both male and female athletes (playing level not specified) [37] or individuals enrolled at military service academies in the USA [41] (Table 1).

**Table 1:** Characteristics of the included studies

Study	Subjects (n)	Age (years) *mean (SD)*	Weight (kg) /height (cm)/BMI (kg/m^**2**^) *mean (SD)*	Activity level	Sport	Functional task	Follow-up	Outcomes	Results	Quality score
Smeets et al. 2019 [42]	ACL injury: 4 females Controls: 35 females	ACL injury: 21 (3) Controls:20.7 (3)	ACL injury: 62.6 (6.9)/172 (10)/ 21.2 (1.0) Controls: 64.9 (7.5)/172 (9)/ 22.1 (1.6)	Elite	Soccer, handball, volleyball	Double-leg vertical drop jump	1 year	3D knee abduction angle (mean across entire landing phase) Peak knee abduction moment	No difference in baseline 3D knee abduction angle or peak knee abduction moment between those who sustained an ACL injury and controls	11/19 (58%)
Räisänen et al. 2018 [37]	ACL injury: 4 females Controls:265 (125 females)	15.7 (1.8)	64.6 (10)/173.3 (9.1)/NR	U21	Floorball, basketball	Single-leg squat	1 year	2D knee abduction angle	NR*	15/19 (79%)
Numata et al. 2018 [40]	ACL injury: 27 females Controls: 27 females	ACL injury: 15 (0) Controls: 15 (0)	ACL injury: 57.4 (7.2)/161.2 (7.1)/ 22.1 (1.5) Controls: 54.3 (5.4)/159.2 (5.8)/ 21.4 (1.9)	High school team sport	Basketball, Handball	Single-leg vertical drop jump	3 years	2D medial displacement (IC, peak, excursion)	Those who sustained an ACL injury had greater baseline MKD at IC and greater peak MKD compared to controls. MKD excursion NR	14/19 (73%)
Krosshaug et al. 2016 [20]	ACL injury: 30 females Controls: 613 females	ACL injury: 19.9 (2.8) Controls: 21.0 (3.9)	ACL injury: 64.6 (7.8)/168 (6.3)/NR Controls: 66.3 (7.9)/169.5 (6.4)/NR	Elite	Soccer, handball	Double-leg vertical drop jump	1-7 years	3D knee abduction angle (IC) 2D medial displacement (excursion) Peak knee abduction moment	No difference in baseline knee abduction at IC, peak knee abduction or MKD between those who sustained an ACL injury and controls	16/19 (84%)
Leppänen et al. 2017 [39]	ACL injury: 15 females Controls*: 327 females	15.4 (1.9)	167.7 (6.2)/60.8 (8.0)/NR	Junior league	Basketball, floorball	Double-leg vertical drop jump	3 years	3D knee abduction angle (IC) 2D medial displacement (excursion) Peak knee abduction moment	No difference between baseline knee abduction at IC, peak knee abduction or knee abduction moment between those who sustained an ACL injury and controls	15/19 (79%)
Dingenen et.al 2015 [38]	ACL injury: 4 females Controls: 40 females	ACL injury: 20.2 (2.9) Controls: 20.8 (3.5)	ACL injury: 61.4 (7.3)/169.1 (10.2)/ 21.4 (1.3) Controls: 64.2 (7.7)/170.7 (8.4)/ 22.1 (2.4)	Elite	Soccer, handball, volley ball	Single-leg vertical drop jump	1 year	2D knee abduction angle	NR	14/19 (73%)
Goerger et al. 2015 [41]	ACL injury: 12 (4 females) Controls: 39 (19 females)	ACL injury: 18.6 (0.5) Controls: 18.5 (0.5)	ACL injury: 72.6 (9.5)/174.1 (7.3)/NR Controls: 70.2 (13.)/172.6 (9.1)/ NR	Military	NA	Double-leg vertical drop jump	3 years	3D knee abduction angle (IC, peak)	No difference between baseline knee abduction at IC, peak knee abduction or knee abduction moment between those who sustained an ACL injury and controls	14/19 (73%)
Nilstad et al. 2014 [35]	ACL injury: 4 females Controls: 134 females	21.5 (4.1)	62 (6) /167 (5)/NR	Elite	Soccer	Double-leg vertical drop jump	1 Year	3D knee abduction angle (peak)	NR	15/19 (79%)
Hewett et al. 2005 [17]	ACL injury: 9 females Controls*: 390 females	ACL injury: 15.8 (1.0) Controls: 16.1 (1.7)	ACL injury: 61.5 (8.3)/167.7 (6.8)/ NR Controls: 59.1 (8.1)/164.1 (6)/ NR	High school team sport	Soccer, basket-ball, volley-ball	Double-leg vertical drop jump	1 Year	3D knee abduction angle (IC, peak) Peak knee abduction moment	Those who sustained an ACL injury had greater baseline knee abduction at IC, peak knee abduction and peak knee abduction moment compared to controls	12/19 (63%)

MKD = medial knee displacement in centimeters, IC = initial contact, * = number of knees, BMI = bodymass index, NR = not reported, * = some studies did not have risk factors for ACL injury as a specific aim and did, thus, not report the result for that variable. For these studies, the data were either retrieved from the authors of that specific study or, were possible, calculated by the authors of this review

### Synthesis of results

**Fig. 2:**
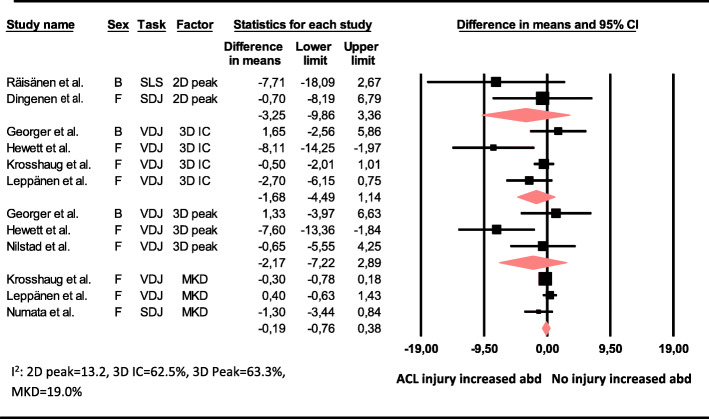
Mean difference in baseline 2D peak knee abduction angle (ACL injury n = 8, controls n = 302), 3D knee abduction angle at initial contact (ACL injury n = 66, controls n = 1369), 3D peak knee abduction angle (ACL injury n = 25, controls n = 563) and medial knee displacement (ACL injury n = 72, controls n = 967) between those who sustained an ACL injury and those who did not. Abd = knee abduction, B = both males and females, F = females, SLS = single-leg squat, SDJ = single-leg drop jump, VDJ = double-leg vertical drop jump, 2D peak = 2D peak knee abduction angle, 3D IC = 3D knee abduction angle at initial contact, 3D peak = 3D peak knee abduction angle, MKD = medial knee displacement

**Fig. 3:**
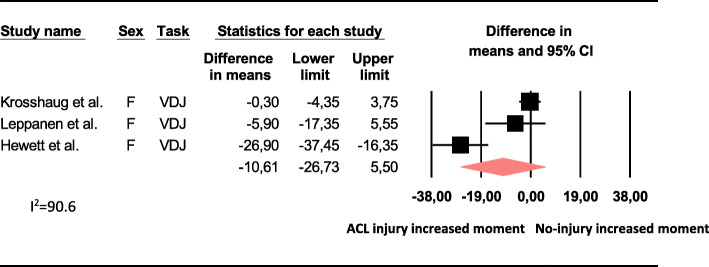
Mean difference in baseline peak knee abduction moment (N.m.) between those who sustained an ACL injury and those who did not (ACL injury *n* = 54, controls *n* = 1330). abd = knee abduction, F = females, VDJ = double-leg vertical drop jump

The meta-analysis showed that there was no difference in baseline 3D knee abduction angle at IC, 3D peak knee abduction angle, 2D peak knee abduction angle, 2D MKD excursion (cm) or peak knee abduction moment between those who subsequently sustained an ACL injury and those who did not (Figs. 2 and 3).

Two articles [40, 42] included factors not eligible for meta-analysis and the results for these articles are reported in Table 2.

**Table 2:** Results of the studies and factors excluded from the meta-analysis

Study	Reason for exclusion of meta-analysis	Results
Smeets et al. 2019 [42]	Only study assessing knee abduction as a mean across the entire landing phase (kinematics) and not reporting sufficient statistics (kinetics)	No difference in 3D knee abduction angle or peak knee abduction moment during a double-leg vertical drop jump between those who sustained an ACL injury (n = 4) and controls (*n* = 35), *p* > 0.05)
Numata et al. 2017 [40]	Only study that reported peak MKD and MKD at initial contact	Those who sustained an ACL injury (n = 27) had greater baseline MKD at IC (mean (sd), 2.1 (2.4) vs 0.4 (2.2) (*p* = 0.006) and greater peak MKD, 8.3 (4.3) vs 5.1 (4.1) (*p* = 0.007) compared to controls (*n* = 27) during a single-leg drop landing

The sensitivity analyses revealed that limiting the studies to those that included only females, or the vertical drop jump task only, or a mean age > 15 years or a follow-up period > 1 year did not change the results (Online resource B).

### Heterogeneity and risk of bias

I^2^ ranged between 13.2 and 90.6%, indicating low to high heterogeneity between studies [43]. The quality of the included studies ranged from 58 to 84%, indicating moderate to high methodological quality (Table 1 and Online resource C, Table 1). There were too few studies in each meta-analysis to explore publication bias using Funnel plots with trim and fill imputations [24].

## Discussion

The result from this systematic review and meta-analysis revealed no association between baseline knee abduction kinematics or kinetics during vertical drop jumps or squats and the risk of sustaining a future ACL injury. No studies were available in other weight-bearing tasks. Our conclusions are based on a large sample (1979 participants across 8 studies), with low to high heterogeneity, and were unaffected in our sensitivity analyses suggesting that our findings hold true irrespective of participant age, sex, or movement task.

A greater knee abduction angle and/or knee abduction moment during weight-bearing activities has commonly been suggested to represent undesirable mechanics and contribute to future ACL injury [6, 8]. Yet, across the 8 studies included in our meta-analyses, we found no difference in 2D knee abduction angle, 3D knee abduction angle, MKD or peak knee abduction moment at baseline between those who sustained a future ACL injury and those who did not. In addition to the possibility that knee abduction kinematics and kinetics are not at all associated with ACL injury risk, one explanation for this apparent contradiction may relate to the magnitude of knee abduction observed in the included studies. The earliest published study to examine the prospective relationship between knee abduction and ACL injury [17], reported that greater knee abduction angle and moment, respectively, were predictive of subsequent ACL injury. In this study, participants that subsequently sustained an ACL injury exhibited ~ 5 degrees of knee abduction at initial contact with the ground, ~ 9 degrees of peak knee abduction and 45 N.m. peak knee abduction moment [17]. Interestingly, all subsequent studies reporting 3D knee abduction mechanics that were included in our meta-analyses report only around 2 degrees of peak abduction for all participants, including those that subsequently sustained an ACL injury [20, 39, 41] and between 21 and 37 N.m. peak abduction moment [20, 39], and found neither measure to be predictive of future ACL injury. Conceivably, the findings of Hewett and colleagues [17], in combination with earlier evidence from cadaver knees [44–46], lead to the development and adoption of ACL injury prevention training specifically targeting knee abduction in weight-bearing activities; this has subsequently been highlighted in numerous reviews and consensus statements [8, 18, 47, 48]. As a result, the magnitude of knee abduction mechanics observed in the vast majority of studies included in our analyses may not be sufficient to present as a risk factor for ACL injury. Supporting this, the study by Krosshaug et.al., [20] included in our analysis reports that approximately 40% of the included participants in their study “reported to have implemented preventive training as part of their routine during the season”. It is, thus, possible that the results of our meta-analyses are rather a consequence of successful injury prevention training in the last decade, than that excessive knee abduction and/or kinetics are not risk factors for ACL injury. On the other hand, although injury prevention programs may have decreased the amount of knee abduction exhibited during activities, there seem to be no decrease in the incidence of ACL injury during the same time period [49, 50], indicating that knee abduction may play a minor role in ACL injury.

An alternative explanation for our findings could be that instead of a linear relationship between knee abduction and ACL injury risk there may be a non-linear relationship with a certain cut-point beyond which knee abduction is associated with ACL injury risk. None of the studies included in this review have used break-point analysis to investigate if certain thresholds of knee abduction were associated with elevated ACL injury risk. Although greater knee abduction has been postulated to increase the risk of injury, there is no consensus regarding the amount of knee abduction that is considered excessive enough to amplify ACL injury risk. Fox et al., determined normative values for knee abduction angle during a vertical drop jump to 0.30 ± 5.0 degrees for IC and 8.71 ± 9.1 degrees for peak knee abduction [51], implying that the participants in the studies included in this review were all in the normal range of knee abduction, i.e., concurrent with the amount of knee abduction in the general population, which may further mask possible associations between knee abduction and injury risk. Given the lack of an injury risk threshold, it is also not clear if there is an elevated risk of knee injury in individuals presenting with knee abduction at the higher end of the normal range that has been postulated. Furthermore, most studies investigating knee abduction as a risk factor for ACL injury assess knee abduction during a drop vertical jump. The vertical drop jump is a bilateral task and may not reflect movements when injury occurs and does not seem to detect sex differences in knee abduction compared to other tasks [18]. Thus, it is possible that this task is not challenging enough to capture the amount of knee abduction that may be associated with injury. Other more challenging tasks, such as cutting tasks, should, therefore, be considered when evaluating knee abduction as a risk factor for ACL injury in future studies.

Increased knee abduction compared to both non-injured individuals and the contra-lateral leg is reported after ACL injury [14–16]. Although several video analysis studies report that knee abduction seems to be involved in the ACL injury mechanism in females [52–54], it is not possible to elucidate the exact time point of the injury on video recordings. Given that the main purpose of the ACL is to provide mechanical stability to the knee [9, 10], it is not clear if the knee abduction (or valgus collapse) observed at the time for injury causes the injury or is due to decreased joint stability as a result of the ACL tear [52–54]. Although some recent cadaveric studies report an association between increased knee abduction moment and ACL failure [55, 56], in support of the latter, a recent systematic review on bone bruises assessed with MRI after ACL injury [57] concludes that knee abduction occurs after the ACL is ruptured, not before. It should, however, be noted that in the same systematic review a high number (approx. 70%) of bone bruises were located on the lateral side, which could indicate presence of knee abduction at the time of injury. Nevertheless, the conclusion of that meta-analysis is further supported by a study that investigated knee kinematics before and after ACL injury and found participants that sustained an ACL injury to perform a drop vertical jump with significantly greater knee abduction angle 2 years after injury compared to their performance at baseline prior to the injury [41]. Thus, it is possible that persistent deficiencies in motor control after injury cause further risk of sustaining also a second ACL injury [20, 31]. It should be noted that although 3D motion analysis was used in most of the studies the way in which knee abduction is quantified may still vary substantially. Differences in how joint axes are defined [58], the kinematic modelling approach employed (direct versus inverse kinematics) [59], and inertial properties used to determine joint kinetics [60] are all known to result in differences in the magnitude of knee abduction measured during functional activities. Similarly, marker placement locations may be differentially influenced by soft tissue artefact, impacting upon the validity and reliability of the marker model used [61]. While there is recent evidence of good to excellent within and between session reliability for both knee abduction angle and knee abduction moment during double-leg vertical drop jump using 3D analysis [62], this may not hold true for all studies included in our review. Despite these variations in the approach used to quantify knee abduction and the variance in the data that this may produce, mostly low to moderate heterogeneity was observed across our meta-analyses, suggesting that the cumulative effect of these differences upon our findings was minimal.

This review has some limitations. We pooled studies on females alone and those that included both men and women, had different follow-up periods as well as different weight-bearing tasks in some of our analyses. While these primary analyses may have masked associations between knee abduction and injury risk, our sensitivity analyses demonstrate that this is unlikely to be the case. Likewise, we pooled studies including participants of different ages (i.e., ≤15 years or > 15 years) and different activity levels. Neuromuscular and biomechanical differences between males and females during early puberty and through maturation have been suggested to play a role for ACL injury risk in young females [8]. Importantly however, our sensitivity analysis including the only two studies on young females (i.e. ≤15 years) revealed no association between 3D knee abduction at baseline and future ACL injury. Taken together, the result from this review applies across sexes, tasks, age and follow-up period. It was, however, not possible to perform a sensitivity analysis for activity level (elite athletes versus high school athletes), since there were too few studies using the same outcome. The two studies that included high school athletes [17, 40] reported that the participants that sustained an ACL injury had increased 3D knee abduction angles (IC and peak) [17] and increased 2D MKD (IC and peak) [40] at baseline compared to those who did not sustain an injury. Thus, we cannot rule out that factors contributing to knee injury may differ between those on an elite level compared to being active on a lower level. This is worthy of further investigation. Furthermore, the meta-analyses are only able to show if a greater or smaller amount of knee abduction is associated with future ACL injury and not if a certain threshold of knee abduction is related to an elevated injury risk. We included studies that employed differing methodologies to quantify knee joint mechanics. Of note, knee abduction angles were obtained with both 2D and 3D motion analysis systems; knee abduction moments were exclusively obtained with 3D motion analysis. While there is evidence that knee abduction angles measured in 2D are strongly correlated with knee abduction measured in 3D [63–65], the 2D measure also incorporates components of sagittal and transverse plane rotation and thus our findings with regard to 2D knee abduction kinematics are likely, to a small extent, to reflect the underlying sagittal and transverse plane knee kinematics. In light of these differences we did not pool the results from 2D and 3D studies. Yet, given the strong relationship between 2D and 3D knee abduction, taken together these results both support the absence of a predictive effect of baseline knee abduction on ACL injury development. Moreover, some of the meta-analysis included a relatively low number of individuals with ACL injury, e.g., the analysis on 2D peak knee abduction (*n* = 8). Performing meta-analysis with a low number of events may increase the risk of overestimating the effect [66]. The 2D peak knee abduction analysis did also include two different tasks, a single-leg squat and a single-leg drop landing with too few studies to perform a sensitivity analysis. Although individuals seem to perform these tasks with a similar amount of knee abduction [67, 68], it is possible that the use of different tasks may have masked findings from individual tasks. Thus, some caution is needed when interpreting the 2D peak knee abduction results. Furthermore, our heterogeneity analysis using I^2^ statistics revealed mostly low to moderate heterogeneity between studies. The analysis for peak knee abduction moment was, however, associated with high heterogeneity. To account for expected heterogeneity, we have performed all analysis under the random effect model that incorporates both within study and between study variance in the analysis. It has also been suggested that the I^2^ statistics may be subject to bias when only a small amount of studies are included in the analysis [69]. Thus, the I^2^ statistics presented in this review should be interpreted with caution. Also, there were too few studies included to be able to explore publication bias. However, since it is more likely that studies reporting no significant results are the studies that are not being published, this is unlikely to have an influence on our result. Finally, this review only included knee abduction kinematics and kinetics as possible risk factors for ACL injury. Several studies highlight that the mechanisms of ACL injury are in fact multifactorial and that several combined factors, such as knee abduction and internal rotation kinematics and kinetics, but also neuromuscular control of the hip and trunk may contribute to the injury mechanism [8, 12, 56, 70–73]. Even though knee abduction kinematics and kinetics alone cannot predict injury risk, future studies will reveal if knee abduction may contribute to knee injury when combined with other risk factors, such as those described above.

## Conclusion

This systematic review and meta-analysis indicates that neither baseline knee abduction kinematics or kinetics during weight-bearing activity may predict future ACL injuries. This is contrary to popular clinical opinion and the findings of the earliest published study examining this relationship. It is possible that as a result of the successful implementation of ACL injury prevention programs in organized sport, emphasizing a knee position in line with the hip and ankle, that knee abduction is not a risk factor for ACL injury development across these cohorts. Future studies are warranted to investigate whether knee abduction during more demanding tasks, in combination with other risk factors and/or in other cohorts, such as recreational athletes, is associated with future primary as well as second ACL injury.

## Supplementary information

**Additional file 1.**

**Additional file 2.**

**Additional file 3.**

## Data Availability

The datasets used and/or analyzed during the current study are available from the corresponding author on reasonable request.

## Abbreviations

ACL: Anterior cruciate ligament
MKD: Medial knee displacement
2D: Two dimensional
3D: Three dimensional
SD: Standard deviation
CI: Confidence intervall
IC: Initial contact
N.m.: Newton meter
MRI: Magnetic resonance imaging
cm: Centimeter
Kg: Kilogram
BMI: Bodymass index
NR: Not reported
abd: Abduction
B: Both males and females
F: Females
SLS: Single-leg squat
SDJ: Single-leg drop jump
VDJ: Double-leg vertical drop jump
