# Supplementary material for: Do knee abduction kinematics and kinetics predict future anterior cruciate ligament injury risk? A systematic review and meta-analysis of prospective studies
Source: BMC Musculoskelet Disord. 2020 Aug 20;21:563. doi: 10.1186/s12891-020-03552-3 (PMC7441716; doi:10.1186/s12891-020-03552-3)
Supplement: Supplementary file 1 — Additional file 1. [file 12891_2020_3552_MOESM1_ESM.docx]

**Online resource A**

**Table 1.** Quality index checklist adapted from Downs and Black

| **ITEM** | | **Yes** | **Unable to determine** | **No** | **N/A** | **Comment** |
| --- | --- | --- | --- | --- | --- | --- |
| 1. | Is the hypothesis/aim/objective of the study clearly described? | □ |  | □ |  |  |
| 2. | Are the main outcomes to be measured clearly described in the Introduction or Methods sections? | □ |  | □ |  |  |
| 3. | Are the characteristics of the subjects included in the study clearly described? | □ |  | □ |  |  |
| 5. | Are the distributions of principle confounders in each group of subjects to be compared clearly described? | □ | □  partially | □ |  |  |
| 6. | Are the main findings of the study clearly described? | □ |  | □ |  |  |
| 7. | Does the study provide estimates of the random variability in the data for the main outcome? | □ |  | □ |  |  |
| 10. | Have actual probability values been reported (e.g. 0.035 rather than <0.05) for the main outcomes except where the probability value is less than 0.01? | □ |  | □ |  |  |
| *External validity* | |  |  |  |  |  |
| 11. | Were the subjects asked to participate in the study representative to the entire population from which they were recruited? | □ | □ | □ |  |  |
| 12. | Where those subjects who were prepared to participate representative of the entire population from which they were recruited? | □ | □ | □ |  |  |
| *Internal validity – Bias* | |  |  |  |  |  |
| 15. | Was an attempt made to blind those measuring the main outcome? | □ | □ | □ |  |  |
| 16. | If any of the results was based on “data dredging“, was this made clear? | □ | □ | □ |  |  |
| 18. | Were the statistical tests used to assess the main outcomes appropriate? | □ | □ | □ |  |  |
| 20. | Were the main outcome measures used accurate (valid and reliable)? | □ | □  Accuracy not reported but method clearly described | □ |  |  |
| *Internal validity – confounding (selection bias)* | |  |  |  |  |  |
| 21. | Were the subjects (e.g. the two groups to be compared) recruited from the same population? | □ | □ | □ |  |  |
| 22. | Were the study subjects (the two groups to be compared) recruited over the same period of time? | □ | □ | □ |  |  |
| 25. | Were there adequate adjustments for confounding in the analyses from which the main findings were drawn? | □ | □ | □ |  |  |
| *Bias* | |  |  |  |  |  |
| 27. | Did the study have sufficient power to detect a clinically important effect? | □ | □ | □ |  |  |

Every question was given 1 point for ”yes” and zero points for ”unable to determine” and ”no” except for item 5 and 20, were 2 points were given for “yes” and 1 point for “partially” and “Accuracy not reported but method clearly described”, respectively. To be able to receive 2 point for item 20, the studies have to report accuracy for all included outcomes.

For studies that did not compare groups i.e. correlation studies, the item 21, 22 and 27 were excluded. Maximum score for group comparison studies were 19 and for correlation studies 16.
