# Supplementary material for: Do knee abduction kinematics and kinetics predict future anterior cruciate ligament injury risk? A systematic review and meta-analysis of prospective studies
Source: BMC Musculoskelet Disord. 2020 Aug 20;21:563. doi: 10.1186/s12891-020-03552-3 (PMC7441716; doi:10.1186/s12891-020-03552-3)
Supplement: Supplementary file 2 — Additional file 2. [file 12891_2020_3552_MOESM2_ESM.docx]

**Online resource B**

**Table 1.** Sensitivity analysis of meta-analytic findings IC=initial contact, MKD=Difference in medial knee displacement in cm between IC and peak

|  | **All studies** | | | **Atypical inclusion criteria (Task)** | | | **Atypical inclusion criteria (Sex)** | | | **Atypical inclusion criteria (age)** | | | **Atypical inclusion criteria**  **(follow-up period <3 years)** | | | **Findings with exclusion of studies** |
| --- | --- | --- | --- | --- | --- | --- | --- | --- | --- | --- | --- | --- | --- | --- | --- | --- |
| **Factor** | Studies  (n) | Participants  (n) | Mean diff | Studies  (n) | Participants  (n) | Mean diff | Studies  (n) | Participants  (n) | Mean diff | Studies  (n) | Participants  (n) | Mean diff | Studies  (n) | Participants  (n) | Mean diff |  |
| 3D knee abduction IC | 4 | 1435 | -1.68  (-4.49 – 1.14) | No studies removed | NA | NA | 3 | 1384 | -2.75  (-6.23-0.74) | 2 | 741 | -4.78  (-9.94 –  0.38) | 3 | 1036 | -0.64 (-2.37 – 1.08) | No changes |
| 3D peak knee abduction | 3 | 588 | -1.99  (-7.83-3.84) | No studies removed | NA | NA | 2 | 537 | -3.55  (-11.62 – 4.55) | 2 | 189 | 0.96  (-2.63 – 4.56) | No studies removed | NA | NA | No changes for sex and age. Insufficient studies retained to perform meta-analyses for follow-up period |
| 2D peak knee abduction | 2 | 310 | -3.06  (-10.2-4.09) | No studies  removed | NA | NA | No studies removed | NA | NA | NA | NA | NA | No studies removed | NA | NA | Insufficient studies retained to perform meta-analyses |
| 2D MKD | 3 | 1039 | -0.20  (-0.86-0.45) | 2 | 985 | -0.10  (-0.10-0.51) | No studies removed | NA | NA | 2 | 396 | -0.32  (-2.11 –  1.49) | No studies removed | NA | NA | No changes |
| Peak knee abduction moment | 3 | 1384 | -10.61  (-26.73 - 5.50) | No studies  removed | NA | NA | No studies  removed | NA | NA | 2 | 771 | -16.52 (-37.10 – 4.06) | 2 | 985 | -0.92  (-4.74  - 2.90) | No changes |
