# Supplementary material for: Do knee abduction kinematics and kinetics predict future anterior cruciate ligament injury risk? A systematic review and meta-analysis of prospective studies
Source: BMC Musculoskelet Disord. 2020 Aug 20;21:563. doi: 10.1186/s12891-020-03552-3 (PMC7441716; doi:10.1186/s12891-020-03552-3)
Supplement: Supplementary file 3 — Additional file 3. [file 12891_2020_3552_MOESM3_ESM.docx]

**Online resource C – risk of bias**

**Table 1.** Quality index scores for the papers included in the systematic review

| **Quality scores** | *Reporting* | | | | | | | *External*  *validity* | | *Internal validity – Bias* | | | | *Internal validity –*  *confounding*  *(selection bias)* | | | | *Power* | **Total score** |
| --- | --- | --- | --- | --- | --- | --- | --- | --- | --- | --- | --- | --- | --- | --- | --- | --- | --- | --- | --- |
|  | Item | | | | | | | | | | | | | | | | | | |
| **Article** | 1 | 2 | 3 | 5 | 6 | 7 | 10 | 11 | 12 | 15 | 16 | 18 | 20 | | 21 | 22 | 25 | 27 |  |
| Smeets et al. 2019 | 1 | 1 | 1 | 1 | 1 | 0 | 0 | 0 | 0 | 0 | 1 | 1 | 2 | | 1 | 1 | 0 | 0 | 11/19  (58%) |
| Räisänen et al. 2018 | 1 | 1 | 1 | 1 | 1 | 1 | 1 | 1 | 0 | 1 | 1 | 1 | 1 | | 1 | 1 | 1 | 0 | 15/19  (79%) |
| Numata et al. 2017 | 1 | 1 | 1 | 1 | 1 | 1 | 1 | 1 | 0 | 0 | 1 | 1 | 2 | | 1 | 1 | 0 | 0 | 14/19  (74%) |
| Krosshaug et al. 2016 | 1 | 1 | 1 | 2 | 1 | 1 | 1 | 1 | 0 | 0 | 1 | 1 | 2 | | 1 | 1 | 1 | 0 | 16/19  (84%) |
| Leppännen et al. 2016 | 1 | 1 | 1 | 2 | 1 | 1 | 1 | 1 | 0 | 0 | 1 | 1 | 1 | | 1 | 1 | 1 | 0 | 15/19  (79%) |
| Dingenen et al. 2015 | 1 | 1 | 1 | 1 | 1 | 1 | 1 | 0 | 0 | 0 | 1 | 1 | 2 | | 1 | 1 | 1 | 0 | 14/19  (74%) |
| Goerger et al. 2015 | 1 | 1 | 1 | 2 | 1 | 1 | 1 | 0 | 0 | 0 | 1 | 1 | 1 | | 1 | 1 | 1 | 0 | 14/19  (74%) |
| Nilstad et al. 2014 | 1 | 1 | 1 | 2 | 1 | 1 | 1 | 1 | 0 | 0 | 1 | 1 | 2 | | 1 | 1 | 0 | 0 | 15/19  (79%) |
| Hewett et al. 2005 | 1 | 1 | 1 | 2 | 1 | 0 | 0 | 0 | 0 | 0 | 1 | 1 | 2 | | 1 | 1 | 0 | 0 | 12/19  (63%) |
